# Effect of Different Time/Temperature Binomials on the Chemical Features, Antioxidant Activity, and Natural Microbial Load of Olive Pomace Paste

**DOI:** 10.3390/molecules28062876

**Published:** 2023-03-22

**Authors:** Maria Manuela Sousa, Diana Melo Ferreira, Susana Machado, Joana C. Lobo, Anabela S. G. Costa, Josman D. Palmeira, Maria Antónia Nunes, Rita C. Alves, Helena Ferreira, Maria Beatriz P. P. Oliveira

**Affiliations:** 1 REQUIMTE/LAQV, Department of Chemical Sciences, Faculty of Pharmacy, University of Porto, R. Jorge de Viterbo Ferreira 228, 4050-313 Porto, Portugal; 2 REQUIMTE/UCIBIO-i4HB, Laboratory of Microbiology, Department of Biological Sciences, Faculty of Pharmacy, University of Porto, R. Jorge de Viterbo Ferreira 228, 4050-313 Porto, Portugal

**Keywords:** olive pomace, food security, heat treatment, vitamin E, fatty acids, hydroxytyrosol

## Abstract

Olive pomace is a by-product from olive oil production that can be further processed to obtain olive pomace paste. In this work, the influence of different time/temperature binomials (65 °C/30 min; 77 °C/1 min; 88 °C/15 s; and 120 °C/20 min) on the nutritional quality, chemical composition, and efficiency on control/elimination of natural microbial load of olive pomace paste was ascertained. The treatments significantly impacted the contents of ash, fat, vitamin E, phenolics (including hydroxytyrosol), flavonoids, and antioxidant activity, but not the fatty acids profile. The binomial 88 °C/15 s showed the greatest potential since it better preserved the phytochemical and antioxidant properties as well as the protein and fiber contents. This binomial is also faster and easy to be implemented at an industrial level, allowing the obtention of a safe functional ingredient to satisfy consumers’ demands for novel sustainable products, simultaneously, responding to food safety and food security concerns.

## 1. Introduction

The world population is expected to reach 9.1 billion people by 2050, implying that 30% more food will be necessary [1]. Consequently, the food industry faces an emergent challenge: to ensure food security while avoiding environmental depletion. A new program called “Transforming our world: the 2030 Agenda for Sustainable Development” aimed at a crucial goal, sustainable food consumption and production [2]. This emphasizes the duty to adopt more sustainable measures, such as adding value to agri-food by-products through the production of healthy food for the growing human population while preventing environmental and natural resources exhaustion.

Portugal was the fourth major olive oil producer in the European Union in 2019 [3]. Therefore, considering that on average 35–40 kg of olive pomace (OP) are produced for each 100 kg of processed olives [4], it is evident that this by-product affects the Portuguese economy and the environment. Three common extraction techniques can be used in olive oil production: traditional pressing mills, three-phase systems, and two-phase systems [4]. Traditional pressing mills are mainly used in small olive mills. In a three-phase system, large amounts of water are added to the olive paste, which leads to the worldwide production of 30 million m^3^ of olive mill wastewater every year. The two-phase system is an eco-friendly system where no water is added. Indeed, in this system, the olive paste is centrifuged, resulting in the production of olive oil and OP. Taking into account that this system reduces wastewater production and allows the obtention of OP, the two-phase system is becoming the most used one [4].

OP is a semi-solid biomass composed of olives’ pulp, skin, and small stone fragments [5]. The interest in this by-product is due to its lipid fraction (rich in α-tocopherol and oleic acid) and its considerable content of bioactive compounds, especially hydroxytyrosol [1]. However, to use this by-product as a fresh ingredient in food products, the remaining stone pieces must be removed, a process that generates a homogenous biomass called olive pomace paste (OPP).

Taking all of this into consideration, in the present work, OP from a two-phase system from Trás-os-Montes, Portugal, was used. The sample was a mixture of the following olive varieties: Cobrançosa, Cordovil, Madural, and Verdeal Transmontana. This work aimed to manually remove the OP’s stone to obtain OPP, characterize the chemical features of OPP, and evaluate the impact of different heat treatments (65 °C/30 min, 77 °C/1 min, 88 °C/15 s, and 120 °C/20 min) on the nutritional quality (proximate analysis, vitamin E and fatty acids (FA) profiles, phytochemicals contents, and antioxidant activity) as well as its efficiency on the control/elimination of the microbiological load of OPP to select the best process for obtaining a functional ingredient for incorporation into foodstuffs to satisfy both consumers’ demands for novel sustainable products and to answer current food security concerns. 

Some previous studies have also evaluated the impact of drying olive pomace through other methods but with slight differences in relation to the present work. Ahmad-Qasem and colleagues (2013) dried olive pomace in a forced air laboratory drier at different temperatures (50, 70, 90, 120, and 150 °C), using a sample from Spain which included the olive pits [6]. Uribe et al., in 2013 and 2014, used a convective dryer at different temperatures (40, 50, 60, 70, 80, and 90 °C), using olive pomace from Chile [7,8]. Uribe et al. (2013) used a mixture of the following olive varieties: Frantoio, Leccino, Racimo, Barnea, and Picual [7], while Uribe and colleagues (2014) used only the Picual olive variety [8]. Pasten and fellow researchers (2019) used a sample of exhausted olive pomace (the remaining olive oil was extracted using solvents) from Chile, which was also dried in a laboratory-scale convective hot-air dryer at 40, 50, 60, 70, 80, and 90 °C [9].

## 2. Results and Discussion

This section presents the results of the proximate analysis, vitamin E and FA profiles, phytochemicals contents, antioxidant activity, and microbiological load of OPP after treatment with different time/temperature binomials. The impact of the heat treatments on the composition of OPP is also discussed. The manual removal of the crushed olive stones from 5.3 kg of OP resulted in 2.3 kg of OPP, so this process had a yield of 43%. Thus, stone pieces make up 57% of OP, a part that contributes to the weight of the samples, but not for analysis as the aim of this study is to obtain a paste for food purposes. Even though this process had low profitability, its escalation into an industrial level is already implemented in some olive mills, e.g., with a stainless sieve and stainless rolls, not to obtain OPP but to recover the stones, which are then sold as biomass. In addition, OPP is a new and alternative approach to use OP, which has been studied by other authors to be handled by drying [6,7,8,9] as mentioned before. The results are expressed both in dry weight (dw) to assess the impact of the different applied treatments, and in fresh weight (fw) to evaluate the quality attributes of this new food ingredient.

### 2.1. Proximate Analysis

OP is a heterogeneous biomass with a moisture content of 50–60 g/100 g and large amounts of minerals, dietary fiber, and oligosaccharides [10]. The results from proximate analysis (Table 1) confirmed that fresh OP had a moisture content of 60.9 g/100 g, also presenting a residual fat content of 1.4 g/100 g (fw), an ash content of 1.1 g/100 g (fw), and a high-fiber content of 17.2 g/100 g (fw). Regarding fresh OPP, a significant 1.2-fold increase in moisture and ash as well as a 1.7-fold increase in total fat, were registered in comparison to the results obtained for fresh OP (*p* < 0.05). However, significant decreases of 56% in carbohydrates and 27% in total fiber were observed, relatively to the fw results. The differences between OP and OPP can be explained by stone removal.

Uribe et al. (2013) showed that, after drying, a loss of protein occurs due to solubility changes or denaturation [7]. Denatured protein is likely to be involved in Maillard reactions, which are reactions that occur between reactive carbonyl groups (reducing sugars) and nucleophilic amino groups (amino acids, peptides, or proteins), resulting in melanoidins formation [7,11]. These reactions occur spontaneously in food exposed to heat, especially if the temperature is above 100 °C [11]. In this study, the applied heat treatments decreased the total protein contents in comparison to OPP (13% in OPPA, 10% in OPPB, 7% in OPPC, and 27% in OPPD, relatively to the dw results). Only OPPD reached temperatures above 100 °C and this was the treatment where the greatest protein loss was detected (*p* < 0.05). On the contrary, the lower impact of OPPA, OPPB, and OPPC on protein content can be explained by the use of temperatures below 100 °C.

The applied heat treatments had a positive impact in the ash content, especially OPPD, where a 1.2-fold increment was observed (*p* < 0.05), regarding the dw results. Total ash increment may be explained by minerals release from the organic matter with the processing temperatures. Indeed, it was reported that processing can enhance the bioaccessibility of some minerals (e.g., Ca and Fe) in vegetable foods [12]. Therefore, it would be interesting to access the individual mineral composition of all samples since OP is rich in minerals, namely, K [7,9,13]. 

OPP residual fat content decreased with the different heat treatments, especially with OPPC and OPPD, which led to a significant loss of 25% and 27%, respectively, regarding the dw results, according to Pasten et al. (2019), temperature could promote fat degradation by hydrolysis or oxidation [9]. 

The results also showed high total fiber contents in all samples (43.5–44.6 g/100 g), but OPP presented the highest amount (48 g/100 g dw). This feature, typical of olive cakes, is related to the presence of olive skin and pulp [8] and confirms that OP is a rich source of dietary fiber [8,9]. The reported healthy properties of fiber in preventing hyperglycemia, decreasing cholesterol levels, reducing colon cancer risk, and heart diseases [7,14], make this fresh heat-treated OPP a product of great interest to the food sector. Regarding the impact of heat treatments in the fiber contents, small but statistically significant decreases were verified, with a maximum loss of 9% (in OPPD), regarding the dw results. Dhingra et al. (2012) reported that processing could change the physicochemical characteristics of dietary fiber [14]. As an example, different heat treatments applied to wheat bran formed heat-resistant fiber-protein complexes [14]. This means that heat treatment is a promising tool to be considered, as it can lead to various changes in food products.

All in all, the use of this heat-treated by-product as a food ingredient could allow the development of foodstuffs with “low-fat” and “high-fiber” claims, considering the proximate analysis results (Table 1) and the UE Regulation (EC) No. 1924/2006 of the European Parliament and Council of 20 December 2006, on nutrition and health claims made on foods. Therefore, this by-product is an undeniably interesting ingredient in answer to the rising interest of consumers in functional foods [15].

### 2.2. Vitamin E Profile

The term “vitamin E” refers to a set of fat-soluble compounds with unique antioxidant properties crucial for health: α-, β-, γ-, and δ-tocopherols, and α-, β-, γ-, and δ-tocotrienols [16]. Total vitamin E content (Table 2) ranged from 4.4 to 6.1 mg/100 g (dw). If we compare the total vitamin E contents in dw of OP and OPP a 1.4-fold increment occurred (*p* < 0.05), which can be explained by stone removal as previously mentioned, and the consequent increase in the total fat content. However, the location of vitamin E vitamers in plant cells should be considered, for example, α-tocopherol is inside chloroplasts; while β- and γ-tocopherols are outside organelles [9]. Therefore, the physical method used to obtain OPP can lead to the rupture of some intact cells and consequent release of a higher quantity of the vitamers. During processing, vitamins are often damaged and lost, due to their susceptibility to environmental factors (e.g., light, temperature, and oxygen), which can affect their stability [17]. This means that food processing can result in vitamin E loss due to exposure to the aforementioned degrading factors. In this study, all heat treatments had a slight negative impact on total vitamin E content (*p* < 0.05), with decreases ranging from 6% to 13% in relation to OPP (dw). Taking these results into consideration and the processing conditions (OPPC was exposed for 15 s to oxygen; while OPPD was processed in a closed glass container), exposure to oxygen may play an essential role in vitamin E degradation, as lower exposure to this factor has resulted in a lower reduction of its amount (only 6% in OPPD). Additionally, vitamin E is a powerful chain-breaking antioxidant [16], protecting long-chain FAs from oxidation [1]. Therefore, the observed reductions in this parameter could also be related to PUFA preservation, which will be discussed in Section 2.3. α-Tocopherol was the major vitamer present in all samples, which was also reported by Nunes et al. (2018) and Pasten et al. (2019) [1,9]. The amounts in fresh samples are similar to the ones reported for other seed oils [18]: guariroba, tamarind, and pinha (1.2, 1.2, and 1.4 mg/100 g, respectively), meaning that this by-product could be an interesting source of α-tocopherol, which is linked to the prevention of lipid peroxidation and scavenging of lipid peroxyl radicals [1]. Despite being heat-stable, α-tocopherol is sensitive to oxidation [7], which can explain the lowest decrease in its content especially in OPPD (7%, dw) in relation to OPP. The processing conditions (minimum oxygen exposure in OPPD) seem to explain these results since α-tocopherol has a considerable susceptibility to oxygen as previously mentioned. The obtained results confirm that OPP is an extremely valuable food ingredient, since their vitamin E contents could promote healthy aging and have potential in preventing cancer, arthritis, and cataracts [16].

### 2.3. Fatty Acids Profile

All samples evidenced a rich composition in oleic acid (73–75%, Table 3), followed by palmitic (11%) and linoleic (9–10%) acids. These results are in agreement with Nunes et al. (2018) and Uribe et al. (2013) [1,7]. In general, OP and OPP had similar FA profiles but some statistical differences were found when comparing monounsaturated fatty acids (MUFA) and polyunsaturated fatty acids (PUFA) sums (*p* < 0.05). A minimal increase in MUFA was observed as well as a slight decrease in PUFA, particularly in linoleic acid. Since PUFA are more vulnerable to lipid peroxidation [19], oxygen exposure during OPP production probably caused their loss. Drying is also a cause of changes in OP’s FA profile since high temperatures can induce lipid hydrolysis or oxidation [9] as stated before. However, in this study, neither the most abundant FAs (oleic, palmitic, and linoleic) nor the MUFA/PUFA ratio were significantly affected by processing. In another study, OP drying also resulted in only minor differences in the FA profile [7]. These results could be explained by the presence of vitamin E that protects PUFA against oxidative damage (Section 2.2). To conclude, the residual fat of this by-product can present noticeable health benefits similar to olive oil: high contents of oleic acid, a FA associated with the inhibition of cholesterol synthesis [9]; and a considerable amount of linoleic acid, a FA linked to blood pressure control [7].

### 2.4. Phytochemicals Contents and Antioxidant Activity

OP contains many functional compounds [10]. Indeed, its high phenolic content makes it phytotoxic [1]. The following phytochemical contents and antioxidant activity assays were accessed to better understand OPP’s phytochemical composition (Table 4).

#### 2.4.1. Total Phenolics Content (TPC)

TPC in olive oil reaches a maximum of 53 mg gallic acid equivalents (GAE)/100 g [20], this is because only 2% of the phenolic compounds pass to olive oil during its production, so 98% remain in OP in two-phase extraction systems [1], which explains the considerable TPC found in OP (3.08 g GAE/100 g dw) in the present study. The production of OPP resulted in an increase of 1.3 times in TPC (dw) explained by stone removal. Pasten et al. (2019) showed that drying could have a negative impact and drying for long periods can lead to phenols aerial oxidation and enzymatic degradation [9]. In this study, all the applied treatments resulted in a slight but significant decrease in the TPC, ranging from 7% to 15% (dw). Additionally, TPC reduction seems to be correlated to oxygen exposure as OPPA and OPPB registered higher losses (15% and 14%, respectively) and were the treatments with prominent oxygen exposure. On the contrary, a minor reduction of 7% was observed in OPPC and OPPD submitted to the minimum oxygen exposure. Kim et al. (2021) reported that heat treatment of apple puree in the presence of oxygen led to a 33% reduction in TPC, while the heat treatment without oxygen preserved the TPC values, allowing to conclude that oxidation may be the cause of phenol degradation, but it can also occur by enzymatic action [21]. Nowadays, the consumption of polyphenol-rich foodstuffs is correlated to the prevention of some diseases, e.g., Parkinson’s, Alzheimer’s, or diabetes [15]. Taking into consideration the considerable TPC of heat-treated OPP and the rising interest for foodstuffs enriched in natural antioxidants, the incorporation of this ingredient in new products has great potential. Indeed, Difonzo et al. (2021) reported that the incorporation of 5% and 10% OP powder in pasta allowed for improved TPC and antioxidant activity results [10].

#### 2.4.2. Total Flavonoids Content (TFC)

Flavonoids are one of the main phenol groups present in olive oil by-products [9], hence the considerable TFC registered in OP (2.69 g catechin equivalents—CE/100 g) (Table 4). Their consumption is linked to a reduction of neuroinflammation, cognition improvement, and weakening of the symptoms of Alzheimer’s [15]. Therefore, the use of fresh heat-treated OPP as a natural food ingredient seems promising. All fresh heat-treated samples registered higher contents than the ones found in other fruits, namely, banana, mango, and mandarin (24.8, 22.7, and 27.4 mg CE/100 g, respectively) [22]. OPP production allowed a 1.3-fold increment in TFC (*p* < 0.05), which can be explained by stones removal and a possible release resulting from OP processing. Flavonoids are also heat-sensitive compounds [23]. In fact, a previous study by Pasten et al. (2019) showed that OP drying resulted in a significant reduction of TFC [9]. Here, all the applied treatments resulted in slight losses (*p* < 0.05), ranging from 3% (OPPD) to 19% (OPPA), regarding the dw results. Moreover, it is noticeable that OPPC and OPPD had the lower impact, being the treatments where oxygen exposure is minimum, whereas the higher oxygen exposure in OPPA and OPPB may explain the lowest contents. These results suggest that oxygen exposure while processing should be minimized, as oxygen seems to be the main degradative factor of the studied antioxidants (vitamin E, TPC, and TFC).

#### 2.4.3. Hydroxytyrosol Content (HTC)

Hydroxytyrosol (HT), the major polyphenol present in OP draws the attention of the food industry due to its health-promoting traits and antioxidant potential [1]. HT is obtained, during olives ripening, from the hydrolysis of oleuropein [5]. Furthermore, during olive crushing and malaxation to produce olive oil, most of the HT glucoside is degraded to HT [24]. The solubility of HT in water [25] and OP’s high moisture content (61 g/100 g) can explain the considerable HTC in all samples (0.35–0.65 g/100 g dw), when compared to the ones found in virgin olive oils: 0.3–29.3 mg/kg [20]. The significant 1.8-fold increase in HTC with OPP production could be explained by stone removal and possible release from cells. HT is one of the most powerful natural antioxidants, and its ability to scavenge reactive species and disrupt peroxidation chain reactions is due to its ortho-diphenolic group [25]. However, its numerous hydroxyl groups are extremely vulnerable to air and light exposure [25]. Therefore, it is possible to foresee that the applied heat-treatments could result in its degradation. In fact, similar to what happened for TPC, a decrease in HTC was registered in all treatments, especially in OPPA with a significant loss of 46% (dw). OPPD had the lowest impact, resulting in a 17% reduction (*p* < 0.05, dw). Once again, the processing conditions may explain the highest impact in OPPA and OPPB since they are more exposed to oxygen. On the other hand, OPPD occurred in a closed vase container, being the sample protected from oxidation, which avoided HT degradation. The same justification can be used for OPPC that was submitted to low-time processing (15 s). HT provides numerous health benefits: cardioprotective, anti-inflammatory, antitumoral, and neuroprotective activities [25]. For example, the enrichment of biscuits with HT allowed a reduction of low-density lipoprotein (LDL) blood levels [26]. Difonzo et al. (2021) reported that a traditional Italian snack showed a higher HTC when it was enriched with 20% fermented OP [10]. Hence, incorporating heat-treated OPP in foodstuffs may provide both healthy and tasty options to consumers.

#### 2.4.4. Ferric Reducing Antioxidant Power (Frap) and 2,2-Diphenyl-1-picrylhydrazyl Radical Scavenging Ability (DPPH^●^-SA)

The antioxidant capacity of all samples was determined by FRAP and DPPH^●^-SA methods. In FRAP assay, 2,4,6-tripyridyl-s-triazine (TPTZ) is reduced to a colored product. In DPPH^●^-SA assay, a radical is neutralized by reduction (electron transference) or quenching (hydrogen transference) [27]. These two methods are often used together due to their complementarity. The considerable FRAP and DPPH^●^-SA values found in OP (4.43 g ferrous sulphate equivalents–FSE/100 g and 1.53 g Trolox equivalents—TE/100 g dw, respectively) are explained by the considerable amounts of phenolics, HT, and flavonoids, which were obtained in the present study and previously discussed. Difonzo et al. (2021) reported that OP incorporation in bread allowed for an increase in the antioxidant capacity [10]. This supports that the antioxidant attributes of this by-product can be exploited in the development of food products, especially those produced to be consumed raw, such as pâtés and toppings, since temperature due to cooking might result in antioxidants loss as seen in the previous results (vitamin E, TPC, TFC, and HTC), which consequently reduces the antioxidant capacity as will be discussed below. Table 4 shows that OPP and OPPD registered the highest values for FRAP and DPPH^●^-SA assays (around 6 g FSE/100 g and 2 g TE/100 g dw, respectively). A significant loss of antioxidant capacity with heat treatments in both assays was registered in this study. These results were expected since antioxidant contents were also reduced due to heat, oxygen, and light sensibility. Pasten et al. (2019) also showed that OP drying resulted in a significant loss of antioxidants [9]. Moreover, Kim et al. (2021) showed that heat-treated apple puree in the presence of oxygen had considerably lower antioxidant activities than fresh apple: FRAP and DPPH^●^-SA methods were reduced 4% and 6%, respectively [21]. Considering the processing conditions of this study, once again, oxygen exposure seems to have the bigger impact on antioxidants since OPPA and OPPB registered the lowest values in both assays. The minimal oxygen exposure can explain the antioxidant capacity registered in OPPD and OPPC in both assays. Nevertheless, there was a slight enhancement of the antioxidant properties in OPPD in FRAP and DPPH^●^-SA methods (fw), which could be explained by the formation of melanoidins, which are a result of Maillard reactions, as significant protein loss was also observed (Section 2.1).

### 2.5. Microbiological Analysis

Olive by-products exhibit antimicrobial activity against pathogenic bacteria and fungi strains due to phenolics’ presence [28]. Therefore, considering the large TPC registered in all samples, OPP could exhibit antimicrobial activity even after a heat treatment. A microbiological analysis was still mandatory to ensure the effectiveness of the applied heat treatments to reduce the natural microbiota of this by-product and, therefore, guarantee consumers’ safety. Indeed, heat treatment is an economic process that aims to reduce or destroy microorganisms, vegetative cells, yeasts, and molds. The total number of microorganisms was counted at 22 °C to understand the diversity of bacteria present at typical ambient temperatures and at 37 °C to encourage the growth of bacteria that can grow at body temperature [29]. According to the results (Table 5), all treatments effectively reduced the natural microbiological load of OPP, especially OPPA, OPPC, and OPPD. The results from OPPA, OPPB, and OPPC were expected to simulate pasteurization, a heat treatment which inactivates the non-spore-forming bacteria and the majority of vegetative spoilage microorganisms [30]. The treatment of OPPB was able to reduce the number of microorganisms in comparison to the control samples, but did not eliminate them entirely in the assay at 37 °C as happened in the other treatments, showing that it was not so effective. The effectiveness of OPPD was also expected since it replicates heat-sterilization, a successful process that kills all forms of microorganisms [31]. To conclude, the applied treatments are of great interest to ensure that OPP meets safety and quality standards and also allows shelf-life extension, being imperative to the sustainability of the food chain supply.

## 3. Materials and Methods

### 3.1. Chemicals

Ultra-pure water was obtained in a Milli-Q purification system (Millipore, Bedford, MA, USA). Chemicals and reagents were of analytical grade. Kjeldahl tablets, absolute ethanol, sodium carbonate (Na_2_CO_3_) decahydrate, sulfuric acid, sodium hydroxide (NaOH), *n*-hexane, and anhydrous sodium sulfate (Na_2_SO_4_) were obtained from Merck (Darmstadt, Germany). HPLC-grade solvents were acquired from Chem-Lab (Zedelgem, Belgium) and Merck (Darmstadt, Germany). The tocopherols and tocotrienols standards and tocol were obtained from Calbiochem (La Jolla, CA, USA) and Matreya Inc. (Pleasant Gap, PA, USA). Folin–Ciocalteus’ reagent, gallic acid, catechin, heptahydrate ferrous sulphate, 2,2-diphenyl-1-picrylhydrazyl radical (DPPH^●^), Trolox, ferric chloride (FeCl_3_), total dietary fiber assay kit, 2,4,6-tripyridyl-s-triazine (TPTZ), sodium nitrite (NaNO_2_), aluminum chloride (AlCl_3_), sodium acetate, boron trifluoride (BF_3_) in methanol solution, butylated hydroxytoluene (BHT), Supelco 37 FAME Mix, and hydroxytyrosol (HT) standard were acquired from Sigma-Aldrich (St. Louis, MI, USA). Boric acid (4%) and potassium hydroxide (KOH) were purchased from Panreac (Barcelona, Spain). Methanol was acquired from Honeywell International, Inc. (Morris Plains, NJ, USA). Sodium chloride (NaCl), chloroform, and sand were acquired from VWR Chemicals (Alfragide, Portugal). Petroleum ether was purchased from Carlo Erba Reagents (Val de Reuil, France). Ethanol (96%) and dichloromethane were acquired from AGA (Prior Velho, Portugal) and Honeywell l Riedel-de Haën TM (Seelze, Germany), respectively. Total microorganism count was performed with Plate Count Agar, Liofilchem (Teramo, Italy).

### 3.2. Sample Preparation

**Figure 1:**
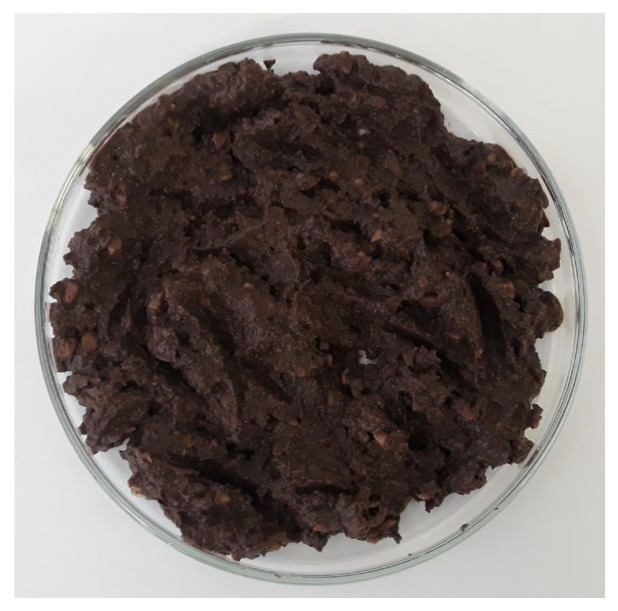
Olive pomace sample.

The sample of OP (5.3 kg, Figure 1) was obtained from a two-phase extraction olive mill (Trás-os-Montes, Portugal). The sample was a mixture of the following olive varieties: Cobrançosa, Cordovil, Madural, and Verdeal Transmontana.

**Figure 2:**
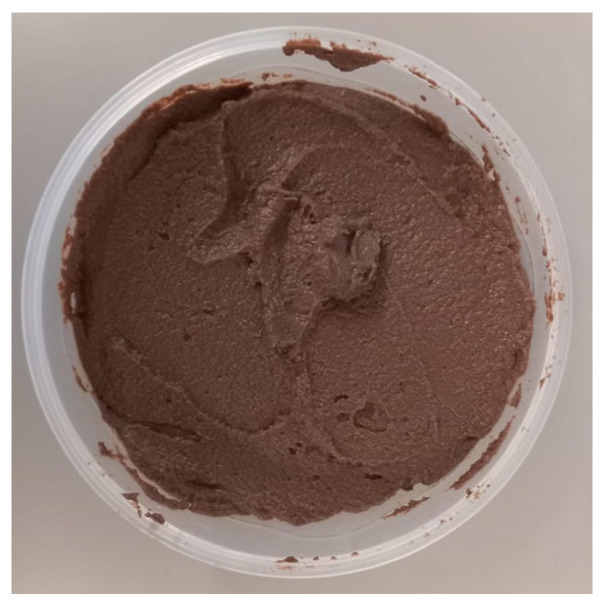
Olive pomace paste.

In the laboratory, after homogenization, the remaining crushed olive stones were manually removed using a stainless-steel sieve to obtain the OPP (Figure 2).

Then, OPP was homogenized and divided into 250 g samples, which were submitted to the different heat treatments (Table 6) to eliminate the natural microbiological load. 

**Figure 3:**
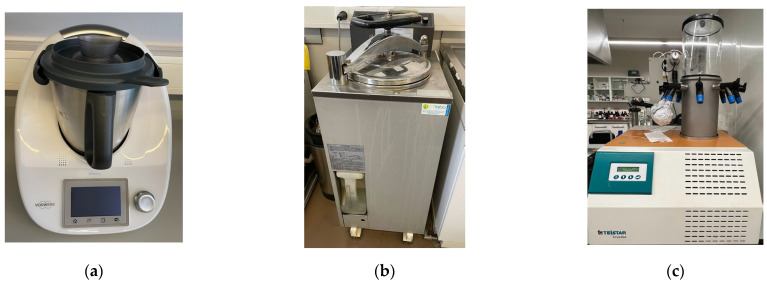
Equipment used for processing the olive pomace paste: (**a**) Thermomix TM 31; (**b**) SANYO Labo autoclave; (**c**) Telstar Cryodos-80 Terrassa.

The designated treatments—OPPA, OPPB, and OPPC—were selected according to Fellows (2009) [32], and were performed in a Thermomix TM 31 (Vorwerk, Wuppertal, Germany, Figure 3a). The OPPD treatment took place in a SANYO Labo autoclave (Gemini BV, Apeldoorn, The Netherlands, Figure 3b). Control samples (OP and OPP) were not submitted to any heat treatment. All six samples (OP, OPP, OPPA, OPPB, OPPC, and OPPD) were stored at −80 °C separately, and lyophilized (Telstar Cryodos-80 Terrassa, Barcelona, Spain, Figure 3c).

### 3.3. Proximate Composition

Proximate analysis was carried out according to official methods [33]: total ash was analyzed in a muffle furnace at 500 °C (920.153 method); total fat was extracted with petroleum ether (991.36 method); total protein was determined by the Kjeldahl method (928.08 method), using 6.25 as the nitrogen conversion factor [34]; and total fiber was assessed by enzymatic-gravimetric procedures (985.29 method). Moisture content was determined in an infrared balance (DBS—KERN & SOHN GmbH, Balingen, Germany). The remaining carbohydrates were calculated by difference [34]. The results are presented in g/100 g of sample, both in fw and dw.

### 3.4. Vitamin E Profile

The lipid fraction of the samples was extracted according to Alves et al. (2009) [35], with minor modifications by Ferreira et al. (2023) [36]. An appropriate amount of sample was weighted to Falcon tubes to obtain 20 mg of fat, then 75 µL of 0.1% BHT solution (m/V), 50 µL of tocol (0.1 mg/mL, internal standard), and 1 mL of absolute ethanol were added. Tubes were stirred for 30 min (Heidolph Multi Reax Vibrating Shaker, VWR International, Radnor, PA, USA). Then, 2 mL of *n*-hexane was added. Tubes were stirred again (30 min) and 1 mL of 1% NaCl solution (*m*/*v*) was added, followed by agitation (2 min) and centrifugation (5000 rpm, 5 min). The supernatant was transferred to new tubes. A re-extraction was performed by adding 2 mL of *n*-hexane, followed by agitation (30 min) and centrifugation (5000 rpm, 5 min). Next, the supernatant was transferred to the second tube and anhydrous Na_2_SO_4_ was added. Tubes were centrifuged (5000 rpm, 5 min), the supernatant was transferred to new tubes, and concentrated under a nitrogen stream until 1 mL. Next, 500 µL of the supernatant was transferred to an amber injection vial for vitamin E profile analysis in a HPLC-DAD-FLD system (Jasco, Tokyo, Japan) equipped with an MD-2015 multiwavelength diode array detector coupled to an FP-2020 fluorescence detector (Jasco, Tokyo, Japan) programmed for excitation and emission at 290 and 330 nm, respectively. Compound separation was accomplished in a normal phase Supelcosil TM LC-SI column (75 mm × 3.0 mm, 3.0 μm, Supelco, Bellefonte, PA, USA). The eluent was 1.2% of 1,4-dioxane in *n*-hexane (*v*/*v*)—isocratic elution. The flow rate and injection volume were 0.7 mL/min and 20 μL, respectively. The UV spectra was used to identify the 8 vitamers previously mentioned. The comparison of their retention times with those of standards was used for quantification, using fluorescence signals and calibration curves obtained with standard stock solutions of each vitamer (α-tocopherol: y = 0.159x − 0.007, R^2^ = 0.9991, 0.22–10.95 μg/mL; β-tocopherol: y = 0.203x − 0.015, R^2^ = 0.9995, 0.21–10.38 μg/mL; γ-tocopherol: y = 0.177x − 0.015, R^2^ = 0.9994, 0.21–13.48 μg/mL; δ-tocopherol: y = 0.226x − 0.018, R^2^ = 0.9995, 0.23–11.71 μg/mL; α-tocotrienol: y = 0.188x − 0.015, R^2^ = 0.9990, 0.19–9.65 μg/mL; β-tocotrienol: y = 0.147x − 0.013, R^2^ = 0.9994, 0.25–12.36 μg/mL; γ-tocotrienol: y = 0.167x − 0.021, R^2^ = 0.9991, 0.24–12.17 μg/mL, and δ-tocotrienol: y = 0.177x + 0.016, R^2^ = 0.9996, 0.24–12.18 μg/mL). The results are presented in μg/100 g of sample in fw and dw.

### 3.5. Fatty Acids Profile

FAs were derivatized to fatty acid methyl esters (FAME), according to ISO 12966-2:2017 [37]. Briefly, the remaining 500 μL of the lipid fraction obtained in Section 3.4. were evaporated under a nitrogen stream and resuspended in 1 mL of dichloromethane. Afterwards, 1.5 mL of 0.5 M KOH in methanol was added, and tubes were stirred and placed in a heating block (100 °C, 10 min, Stuart SBH130D/3 block heater, Stafford, UK). After cooling (ice, 5 min), 2 mL of 14% BF_3_ in methanol was added followed by another heating (100 °C, 30 min) and cooling (ice, 5 min) period. After adding 2 mL of deionized water and 4 mL of *n*-hexane, tubes were centrifuged (3000 rpm, 5 min). The supernatant was mixed with anhydrous Na_2_SO_4_, centrifuged (3000 rpm, 5 min), and 1 mL was transferred to injection vials for FA profile analysis in a gas chromatograph coupled to a flame ionization detector (GC-FID, Shimadzu GC-2010 Plus, Shimadzu, Tokyo, Japan) and a split/splitless AOC-20i auto-injector (Shimadzu, Tokyo, Japan). FAME separation was achieved in a CP-Sil 88 silica capillary column (50 m × 0.25 mm i.d.; 0.20 µm film thickness, Varian, Middelburg, The Netherlands), using helium as carrier gas. The temperature program used was: 120 °C for 5 min; increase to 160 °C at 2 °C/min; hold for 15 min; and increase to 220 °C at 2 °C/min. Injector and detector were at 250 °C and 270 °C, respectively. A split ratio of 1:50 and an injection volume of 1.0 μL were used. FAME identification was carried out by comparing their retention times with those of standards (Supelco 37 Component FAME Mix, Supelco, Bellefonte, PA, USA). Data were analyzed based on relative peak areas, being the results expressed in relative % of each FA.

### 3.6. Phytochemicals Contents and Antioxidant Activity

For extracts preparation, 125 mg of each sample was mixed with 50 mL of methanol and deionized water (80/20; *v*/*v*) solution, at 40 ± 2 °C, under constant stirring (600 rpm, 60 min, MS-H-S10 magnetic stirrer, ChemLand, Stargard, Poland), in triplicate. Extracts were filtered (Whatman No. 4 filter paper) and stored at −20 °C until further analysis.

#### 3.6.1. Total Phenolics Content (TPC)

TPC was determined according to Ferreira et al. (2023) [36]. In a microplate, 30 µL of each extract (Section 3.6) was mixed with 150 µL of Folin–Ciocalteu’s reagent (1:10) and 120 µL of 7.5% (m/V) Na_2_CO_3_. The microplate was incubated at 45 °C for 15 min, followed by 30 min at room temperature (RT), light-protected. The sample’s absorbance was measured in a microplate reader (Synergy HT GENS5, BioTek Instruments, Inc., Winooski, VT, USA) at 765 nm. A gallic acid calibration curve (y = 0.009x + 0.006, R^2^ = 0.999, 5–100 mg/L) was used for quantification. Results are expressed in g of GAE/100 g of sample in fw and dw.

#### 3.6.2. Total Flavonoids Content (TFC)

TFC was evaluated according to Ferreira et al. (2023) [36]. Thus, 1 mL of each extract (Section 3.6) was mixed with 4 mL of deionized water and 300 µL of 5% NaNO_2_ (m/V). After 5 min at RT, 300 µL of 10% AlCl_3_ (*m*/*v*) was added to the previous mixture. Later, after incubation at RT (1 min), 2 mL of 1 M NaOH and 2.5 mL of deionized water were added. The absorbance was measured at 510 nm in a microplate reader (Synergy HT GENS5, BioTek Instruments, Inc., Winooski, VT, USA). A catechin calibration curve (y = 0.002x + 0.001, R^2^ = 0.998, 2.5–400 mg/L) was used for quantification. The sample’s TFC is expressed in g of CE/100 g of sample in fw and dw.

#### 3.6.3. Hydroxytyrosol Content (HTC)

HT analysis was carried out with 1 mL of each extract (Section 3.6) in an HPLC-DAD-FLD system (Jasco, Tokyo, Japan), consisting of a LC-NetII/ADC hardware interface, a pump (Jasco PU-2089), an automatic sampler (Jasco AS-2057 Plus), a multiwavelength diode array detector (Jasco MD-2018 Plus) coupled to a fluorescence detector (Jasco FP-2020 Plus) and a column thermostat (Jasco CO-2060 Plus). HT was evaluated by fluorescence and monitored at λ excitation and λ emission of 280 and 330 nm, respectively. A gradient elution program using as solvents acetic acid (A, 1%) and methanol (B, 100%) was employed: 0 min, 5% B; 30 min, 25% B; 50 min, 75% B; 55 min, 100% B; 60 min, 100% B; 63 min, 5% B. A Zorbax-SB-C18 (250 × 4.6 mm, 5 μm, Agilent Technologies, Amstelveen, The Netherlands) chromatographic column was used, at 20 °C, with a flow rate of 1 mL/min and an injection volume of 20 µL. A HT calibration curve was obtained (y = 10147x + 3486.5, R^2^ = 0.9998, 0.25–200 μg/mL). Results are presented in g/100 g of sample in fw and dw.

#### 3.6.4. Ferric Reducing Antioxidant Power (FRAP) and 2,2-Diphenyl-1-picrylhydrazyl Radical Scavenging Ability (DPPH^●^-SA)

FRAP assay was determined according to Ferreira et al. (2023) [36]. In a microplate, aliquots of 35 µL of each extract (Section 3.6) were mixed with 265 µL of the FRAP reagent: acetate buffer (0.3 M), TPTZ solution (10 mM), and FeCl_3_ (20 mM). The microplate was incubated (37 °C, 30 min), light-protected, and the absorbance was measured in a microplate reader at 595 nm (Synergy HT GENS5, BioTek Instruments, Inc., Winooski, VT, USA). A calibration curve with ferrous sulphate (y = 0.002x + 0.080, R^2^ = 0.999, 25–500 mg/L) was used for quantification. Results were expressed as g of FSE/100 g of sample in fw and dw.

DPPH^●^-SA assay was determined according to Ferreira et al. (2023) [36]. In a microplate, aliquots of 30 µL of each extract (Section 3.6) were mixed with 270 µL of fresh DPPH^●^ solution in ethanol (6 × 10^−2^ mM). Absorbance (525 nm) was measured in a microplate reader (Synergy HT GENS5, BioTek Instruments, Inc., Winooski, VT, USA) every 2 min until reaction endpoint at 20 min to assess the kinetics of the reaction. A Trolox calibration curve was obtained (y = −0.007x + 0.540, R^2^ = 0.999, 5.62–175.34 mg/L) and results presented in g of TE/100 g of sample in fw and dw.

### 3.7. Microbiological Analysis

The samples were serially diluted until 10^−8^ dilution, with ultrapure sterile water. Then, in all dilutions (from 10^−1^ to 10^−8^), the total count of microorganisms was determined to evaluate the efficacy of the applied processes in the microbiological load reduction. The total count of microorganisms was achieved by the pour-plate method, meaning that 1 mL of each sample dilution was poured into different sterile Petri plate dishes, and then mixed with 20 mL of liquid plate count agar (PCA) cooled to about 50 °C. After solidification, plates were incubated at two different temperatures (22 °C and 37 °C) for 48 h (*n* = 3). In the analysis of the results, only the visual counting of the colony-forming units (CFU) that yield between 30 and 300 CFU was considered. The results obtained for the different heat-treatments (OPPA, OPPB, OPPC, and OPPD) were compared with OP and OPP (without treatment) to assess the efficiency of the applied treatments in the reduction of microbiological load. The results were expressed as the mean of CFU for each incubation temperature.

### 3.8. Statistical Analysis

Statistical analysis was performed using IBM SPSS v. 25 (IBM Corp., Armonk, NY, USA). Data are expressed as mean ± standard deviation. Significant differences between samples were assessed by one-way ANOVA, followed by Tukey’s HSD to make pairwise comparisons between means. The level of significance for all hypothesis tests (*p*) was 0.05.

## 4. Conclusions

OPP (obtained from OP, a by-product of olive oil production) presented high moisture, residual fat, and considerable fiber contents. Moreover, its lipid fraction can be considered a quality attribute as a source of vitamin E (especially α-tocopherol), oleic acid (a MUFA related to healthy characteristics and food stability), and linoleic acid (a PUFA known for reducing total and LDL cholesterol blood levels). Additionally, it showed high contents of total phenolics, particularly hydroxytyrosol, and total flavonoids, allowing the development of food products with natural antioxidant properties. All of this is in agreement with new consumer trends: the search for functional foods rich in bioactive compounds.

In this study, OPP was submitted to four different heat treatments. All effectively reduced the microbial load, meaning that the use of heat-treated OPP as a functional ingredient will not compromise consumers’ health. Overall, the selected treatments had a negative impact on the quality attributes of OPP, especially in vitamin E, namely, α-tocopherol, total phenolics, particularly hydroxytyrosol, total flavonoids, and antioxidant capacity. Nevertheless, none of them significantly affected OPP’s FA profile, which remained a source of beneficial FA (oleic and linoleic acids). 

An interesting conclusion of this study is that the heat treatment should be carefully planned and evaluated, especially in relation to oxygen exposure, while processing food products to maintain their nutritional quality. Indeed, undesirable changes in the antioxidant quality occurred with heat-treatment that can be related to the presence of oxygen. Therefore, oxygen exposure should be limited.

From the applied heat-treatments, OPPC (88 °C/15 s) and OPPD (120 °C/20 min) stood out due to the lower impact on the previously mentioned bioactive compounds and antioxidant properties. However, foreseeing the sustainable development and the cost of processing, OPPC was selected as the best treatment to apply for this product at an industrial level for further incorporation into foodstuffs as it is the fastest method and completely eliminated the microbiological load.

To conclude, even after heat treatment, OPP remains an interesting nutritional biomass due to its contents of total dietary fiber, vitamin E, and other bioactives. Therefore, incorporating heat-treated OPP at 88 °C for 15 s in new functional food products seems promising due to the health-related characteristics. Moreover, it contributes to the circular economy and sustainability of the olive oil sector, since it allows the use of OP, a by-product that otherwise represents an environmental burden.

## Figures and Tables

**Table 1:** Proximate composition of olive pomace samples.

g/100 g	Sample	Moisture	Total Protein	Ash	Total Fat	Total Fiber	Remaining Carbohydrates
Dry weight	OP	-	6.3 ± 0.8 ^c^	2.7 ± 0.0 ^d^	3.6 ± 0.1 ^c^	44.0 ± 0.9 ^b^	43.4 ± 1.6 ^a^
	OPP	-	9.6 ± 0.6 ^a^	4.9 ± 0.0 ^c^	9.3 ± 0.3 ^a^	48.0 ± 0.9 ^a^	28.2 ± 1.8 ^c^
	OPPA	-	8.4 ± 0.2 ^ab^	5.3 ± 0.0 ^bc^	8.1 ± 0.4 ^ab^	44.0 ± 0.4 ^b^	34.1 ± 0.2 ^b^
	OPPB	-	8.6 ± 0.5 ^ab^	5.4 ± 0.0 ^b^	8.2 ± 0.2 ^ab^	44.3 ± 0.1 ^b^	33.5 ± 0.8 ^bc^
	OPPC	-	8.9 ± 0.1 ^ab^	5.2 ± 0.0 ^bc^	7.0 ± 0.0 ^b^	44.6 ± 0.4 ^b^	34.4 ± 0.4 ^b^
	OPPD	-	7.0 ± 0.6 ^bc^	5.9 ± 0.3 ^a^	6.8 ± 0.9 ^b^	43.5 ± 0.2 ^b^	36.8 ± 2.1 ^b^
Fresh weight	OP	60.9 ± 0.3 ^d^	2.5 ± 0.3 ^a^	1.1 ± 0.0 ^d^	1.4 ± 0.0 ^c^	17.2 ± 0.3 ^a^	16.9 ± 0.3 ^a^
	OPP	73.9 ± 0.1 ^ab^	2.5 ± 0.2 ^a^	1.3 ± 0.0 ^c^	2.4 ± 0.1 ^a^	12.5 ± 0.2 ^b^	7.4 ± 0.4 ^d^
	OPPA	73.1 ± 0.0 ^bc^	2.2 ± 0.1 ^a^	1.4 ± 0.0 ^b^	2.2 ± 0.1 ^ab^	11.8 ± 0.1 ^bc^	9.2 ± 0.1 ^bc^
	OPPB	74.7 ± 0.3 ^a^	2.2 ± 0.1 ^a^	1.4 ± 0.0 ^bc^	2.1 ± 0.0 ^ab^	11.2 ± 0.0 ^c^	8.5 ± 0.5 ^cd^
	OPPC	73.9 ± 0.6 ^ab^	2.3 ± 0.0 ^a^	1.3 ± 0.0 ^bc^	1.8 ± 0.0 ^bc^	12.3 ± 0.1 ^b^	8.3 ± 0.5 ^cd^
	OPPD	72.4 ± 0.0 ^c^	1.9 ± 0.2 ^a^	1.6 ± 0.1 ^a^	1.9 ± 0.3 ^bc^	11.4 ± 0.1 ^c^	10.8 ± 0.6 ^b^

OP, olive pomace; OPP, olive pomace paste; OPPA, olive pomace paste processed at 65 °C, 30 min; OPPB, olive pomace paste processed at 77 °C, 1 min; OPPC, olive pomace paste processed at 88 °C, 15 s; OPPD, olive pomace paste processed at 120 °C, 20 min. The results are presented in g/100 g of sample in fresh or dry weight, as mean ± standard deviation (*n* = 3). Within each column, different letters represent significant differences (*p* < 0.05) between samples, for results expressed in fresh or dry weight, separately.

**Table 2:** Vitamin E profile of olive pomace samples.

μg/100 g	Sample	α-tocopherol	α-tocotrienol	β-tocopherol	γ-tocopherol	δ-tocopherol	Total Vitamin E
Dry weight	OP	4133 ± 138 ^d^	62 ± 1 ^c^	50 ± 1 ^c^	97 ± 2 ^c^	17 ± 0 ^c^	4360 ± 143 ^d^
	OPP	5715 ± 227 ^a^	109 ± 1 ^b^	78 ± 2 ^b^	162 ± 6 ^ab^	37 ± 0 ^b^	6101 ± 234 ^a^
	OPPA	4906 ± 65 ^c^	130 ± 1 ^a^	73 ±1 ^b^	146 ± 4 ^b^	36 ± 0 ^b^	5290 ± 65 ^c^
	OPPB	4983 ± 84 ^c^	127 ± 5 ^a^	73 ± 1 ^b^	146 ± 3 ^b^	37 ± 0 ^b^	5365 ± 87 ^c^
	OPPC	5066 ± 42 ^bc^	112 ± 3 ^b^	71 ± 0 ^b^	148 ± 5 ^b^	32 ± 0 ^b^	5430 ± 45 ^bc^
	OPPD	5335 ± 93 ^b^	nd	106 ± 6 ^a^	165 ± 11 ^a^	115 ± 4 ^a^	5722 ± 112 ^b^
Fresh weight	OP	1614 ± 54 ^a^	24 ± 0 ^d^	20 ± 1 ^b^	38 ± 1 ^bc^	7 ± 0 ^c^	1703 ± 56 ^a^
	OPP	1489 ± 59 ^b^	28 ± 0 ^c^	20 ± 1 ^b^	42 ± 2 ^ab^	10 ± 0 ^b^	1590 ± 61 ^b^
	OPPA	1318 ±17 ^c^	35 ± 0 ^a^	20 ± 0 ^b^	39 ± 1 ^bc^	10 ± 0 ^b^	1421 ± 18 ^c^
	OPPB	1262 ± 21 ^c^	32 ± 1 ^b^	18 ± 0 ^b^	37 ± 1 ^c^	9 ± 0 ^b^	1358 ± 22 ^c^
	OPPC	1324 ± 11 ^c^	29 ± 1 ^c^	18 ± 0 ^b^	39 ± 1 ^bc^	8 ± 0 ^b^	1419 ± 12 ^c^
	OPPD	1472 ± 26 ^b^	nd	29 ± 2 ^a^	46 ± 3 ^a^	32 ± 1 ^a^	1579 ± 31 ^b^

OP, olive pomace; OPP, olive pomace paste; OPPA, olive pomace paste processed at 65 °C, 30 min; OPPB, olive pomace paste processed at 77 °C, 1 min; OPPC, olive pomace paste processed at 88 °C, 15 s; OPPD, olive pomace paste processed at 120 °C, 20 min; nd, not detected. The results are presented in μg/100 g of sample in fresh or dry weight, as mean ± standard deviation (*n* = 3). Within each column, different letters represent significant differences (*p* < 0.05) between samples, for results expressed in fresh or dry weight, separately.

**Table 3:** Fatty acids profile of olive pomace samples.

Fatty Acids (Relative %)	OP	OPP	OPPA	OPPB	OPPC	OPPD
Myristic (C14:0)	0.03 ± 0.00 ^a^	0.02 ± 0.00 ^b^	0.02 ± 0.00 ^b^	0.02 ± 0.00 ^b^	0.03 ± 0.00 ^a^	0.03 ± 0.00 ^a^
Palmitic (C16:0)	11.18 ± 0.08 ^a^	11.18 ± 0.04 ^a^	11.24 ± 0.01 ^a^	11.30 ± 0.15 ^a^	11.24 ± 0.02 ^a^	11.25 ± 0.02 ^a^
Palmitoleic (C16:1)	0.59 ± 0.03 ^a^	0.60 ± 0.03 ^a^	0.63 ± 0.00 ^a^	0.60 ± 0.03 ^a^	0.63 ± 0.01 ^a^	0.64 ± 0.00 ^a^
Heptanoic (C17:0)	0.10 ± 0.00 ^a^	0.10 ± 0.00 ^a^	0.10 ± 0.00 ^a^	0.10 ± 0.01 ^a^	0.10 ± 0.00 ^a^	0.10 ± 0.00 ^a^
Stearic (C18:0)	2.82 ± 0.15 ^a^	2.85 ± 0.20 ^a^	2.79 ± 0.01 ^a^	2.84 ± 0.21 ^a^	2.81 ± 0.01 ^a^	2.79 ± 0.04 ^a^
Oleic (C18:1n9c)	73.07 ± 0.40 ^b^	74.69 ± 0.26 ^a^	74.37 ± 0.12 ^a^	74.41 ± 0.11 ^a^	74.29 ± 0.03 ^a^	74.24 ± 0.24 ^a^
Linoleic (C18:2n6c)	9.97 ± 0.47 ^a^	8.52 ± 0.33 ^b^	8.79 ± 0.13 ^b^	8.68 ±0.28 ^b^	8.85 ± 0.05 ^b^	8.91 ± 0.07 ^b^
Arachidic (C20:0)	0.51 ± 0.05 ^a^	0.47 ± 0.03 ^a^	0.45 ± 0.00 ^a^	0.46 ± 0.03 ^a^	0.46 ± 0.01 ^a^	0.45 ± 0.04 ^a^
α-linolenic (C18:3n3)	0.92 ± 0.10 ^a^	0.90 ± 0.10 ^a^	0.96 ± 0.01 ^a^	0.89 ± 0.07 ^a^	0.93 ± 0.02 ^a^	0.93 ± 0.02 ^a^
*cis*-11-Eicosenoic (C20:1n9)	0.38 ± 0.01 ^a^	0.34 ± 0.02 ^a^	0.35 ± 0.00 ^a^	0.35 ± 0.01 ^a^	0.35 ± 0.01 ^a^	0.35 ± 0.02 ^a^
Behenic (C22:0)	0.28 ± 0.04 ^a^	0.21 ± 0.02 ^b^	0.21 ± 0.00 ^b^	0.22 ± 0.03 ^ab^	0.21 ± 0.00 ^b^	0.20 ± 0.02 ^b^
Lignoceric (C24:0)	0.16 ± 0.02 ^a^	0.12 ± 0.01 ^b^	0.11 ± 0.00 ^b^	0.12 ± 0.00 ^b^	0.11 ± 0.01 ^b^	0.12 ± 0.01^b^
∑ SFA	15.07 ± 0.15 ^a^	14.97 ± 0.22 ^a^	14.91 ± 0.01 ^a^	15.07 ± 0.35 ^a^	14.95 ± 0.03 ^a^	14.93 ± 0.12 ^a^
∑ PUFA	10.89 ± 0.46 ^a^	9.42 ± 0.34 ^b^	9.74 ± 0.10 ^b^	9.57 ± 0.28 ^b^	9.78 ± 0.05 ^b^	9.85 ± 0.07 ^b^
∑ MUFA	74.03 ± 0.31 ^b^	75.64 ± 0.20 ^a^	75.34 ± 0.10 ^a^	75.36 ± 0.10 ^a^	75.27 ± 0.02 ^a^	75.22 ± 0.18 ^a^
MUFA/PUFA	6.81 ± 0.33 ^b^	8.04 ± 0.31 ^a^	7.73 ± 0.09 ^a^	7.88 ± 0.23 ^a^	7.70 ± 0.04 ^a^	7.64 ± 0.07 ^a^

OP, olive pomace; OPP, olive pomace paste; OPPA, olive pomace paste processed at 65 °C, 30 min; OPPB, olive pomace paste processed at 77 °C, 1 min; OPPC, olive pomace paste processed at 88 °C, 15 s; OPPD, olive pomace paste processed at 120 °C, 20 min; SFA, saturated fatty acids; MUFA, monounsaturated fatty acids; PUFA, polyunsaturated fatty acids. The results are expressed in relative % as mean ± standard deviation (*n* = 3) in dry weight. Within each line, different letters represent significant differences (*p* < 0.05) between samples.

**Table 4:** Phytochemicals contents and antioxidant activity of olive pomace samples.

	Sample	TPC g GAE/100 g	TFC g CE/100 g	HTC g/100 g	FRAP g FSE/100 g	DPPH^●^-SA g TE/100 g
Dry weight	OP	3.08 ± 0.13 ^d^	2.69 ± 0.03 ^d^	0.36 ± 0.00 ^cd^	4.43 ± 0.57 ^d^	1.53 ± 0.06 ^bc^
	OPP	4.09 ± 0.11 ^a^	3.44 ± 0.03 ^a^	0.65 ± 0.04 ^a^	6.10 ± 0.28 ^a^	1.84 ± 0.10 ^a^
	OPPA	3.46 ± 0.14 ^c^	2.80 ± 0.19 ^cd^	0.35 ± 0.02 ^d^	4.98 ± 0.11 ^c^	1.38 ± 0.05 ^c^
	OPPB	3.50 ± 0.16 ^c^	2.88 ± 0.09 ^c^	0.40 ± 0.03 ^cd^	5.17 ± 0.17 ^bc^	1.46 ± 0.04 ^c^
	OPPC	3.81 ± 0.15 ^b^	3.10 ± 0.08 ^b^	0.42 ± 0.02 ^c^	5.47 ± 0.26 ^b^	1.66 ± 0.12 ^b^
	OPPD	3.81 ± 0.13 ^b^	3.32 ± 0.20 ^a^	0.54 ± 0.03 ^b^	6.10 ± 0.43 ^a^	1.92 ± 0.14 ^a^
Fresh weight	OP	1.20 ± 0.05 ^a^	1.05 ± 0.01 ^a^	0.14 ± 0.00 ^b^	1.73 ± 0.22 ^a^	0.60 ± 0.02 ^a^
	OPP	1.07 ± 0.03 ^b^	0.90 ± 0.01 ^b^	0.17 ± 0.01 ^a^	1.59 ± 0.07 ^a^	0.48 ± 0.03 ^c^
	OPPA	0.93 ± 0.04 ^d^	0.75 ± 0.05 ^d^	0.09 ± 0.00 ^c^	1.34 ± 0.03 ^b^	0.37 ± 0.01 ^d^
	OPPB	0.89 ± 0.04 ^d^	0.73 ± 0.02 ^d^	0.10 ± 0.01 ^c^	1.31 ± 0.04 ^b^	0.37 ± 0.01 ^d^
	OPPC	1.00 ± 0.04 ^c^	0.81 ± 0.02 ^c^	0.11 ± 0.00 ^c^	1.43 ± 0.07 ^b^	0.43 ± 0.03 ^c^
	OPPD	1.05 ± 0.04 ^bc^	0.92 ± 0.05 ^b^	0.15 ± 0.01 ^ab^	1.68 ± 0.12 ^a^	0.53 ± 0.04 ^b^

OP, olive pomace; OPP, olive pomace paste; OPPA, olive pomace paste processed at 65 °C, 30 min; OPPB, olive pomace paste processed at 77 °C, 1 min; OPPC, olive pomace paste processed at 88 °C, 15 s; OPPD, olive pomace paste processed at 120 °C, 20 min; TPC, total phenolics content; TFC, total flavonoids content; HTC, hydroxytyrosol content; FRAP, ferric reducing antioxidant power; DPPH^●^-SA, 2,2-diphenyl-1-picrylhydrazyl radical scavenging ability; GAE, gallic acid equivalents; CE, catechin equivalents; FSE, ferrous sulphate equivalents; TE, trolox equivalents. The results are presented as mean ± standard deviation (*n* = 3). Within each column, different letters represent significant differences (*p* < 0.05) between samples, for results expressed in fresh or dry weight, separately.

**Table 5:** Total count of microorganisms at 22 °C and 37 °C (48 h) of olive pomace samples.

Temperature	Sample	Dilution	Total Count of Microorganisms (CFU)
22 °C	OP	10^−2^	3.6 × 10^3^
	OPP	10^−2^	4.4 × 10^3^
	OPPA	10^−1^	Ø
	OPPB	10^−1^	Ø
	OPPC	10^−1^	Ø
	OPPD	10^−1^	Ø
37 °C	OP	10^−1^	1.2 × 10^3^
	OPP	10^−1^	1.2 × 10^3^
	OPPA	10^−1^	Ø
	OPPB	10^−1^	5.3 × 10^2^
	OPPC	10^−1^	Ø
	OPPD	10^−1^	Ø

OP, olive pomace; OPP, olive pomace paste; OPPA, olive pomace paste processed at 65 °C, 30 min; OPPB, olive pomace paste processed at 77 °C, 1 min; OPPC, olive pomace paste processed at 88 °C, 15 s; OPPD, olive pomace paste processed at 120 °C, 20 min; CFU, colony forming unit; Ø, result below 30 CFU.

**Table 6:** Time/temperature binomials applied to the olive pomace paste (OPP).

Samples	Temperature (°C)	Time
OPPA	65	30 min
OPPB	77	1 min
OPPC	88	15 s
OPPD	120	20 min

## Data Availability

Not applicable.

## Sample Availability

Samples of olive pomace are not available from the authors upon request, but they can be provided by the olive oil producer during the seasonal harvest period.
